# Nm23-H1核内定位对人肺腺癌A549细胞增殖的影响

**DOI:** 10.3779/j.issn.1009-3419.2017.04.02

**Published:** 2017-04-20

**Authors:** 亚 盛, 艳丽 熊, 明芳 许, 勋杰 况, 东 王, 雪琴 杨

**Affiliations:** 400042 重庆，第三军医大学大坪医院野战外科研究所肿瘤中心 Cancer Center, Daping Hospital and Research Institute of Surgery, Third Military Medical University, Chongqing 400042, China

**Keywords:** 肺肿瘤, Nm23-H1, 核内定位, 细胞周期, 细胞增殖, Lung neoplasms, Nm23-H1, Nuclear localization, Cell cycle, Cell proliferation

## Abstract

**背景与目的:**

现有研究发现Nm23-H1还存在胞核表达，而既往的研究都是以过表达或抑制胞浆Nm23-H1为研究手段，由于Nm23-H1本身缺乏核引导序列，其研究结果并不能真实反映或重复临床中Nm23-H1以胞核定位为主的实际生物学效应。因此，本研究通过构建带有核引导序列的Nm23-H1载体并转染A549细胞以探讨Nm23-H1从胞浆向胞核转位对肺癌细胞增殖的影响。

**方法:**

采用基因重组技术构建带核定位信号序列的pLentis-CMV-NME1-IRES2-PURO慢病毒载体，酶切和测序鉴定正确后，稳定转染A549细胞后用Western blot和激光共聚焦检测Nm23-H1蛋白的定位和表达，用CCK-8法检测细胞的增殖，流式细胞术检测细胞周期变化。

**结果:**

成功构建了核内定向表达Nm23-H1的慢病毒载体。转染组在72 h、96 h和120 h时增殖率与空载体组相比均显著升高（*P* < 0.000, 1）。空载体组A549细胞在G_0_期/G_1_期所占比例为35.69%，高于转染组的28.28%（*t*=1.461, *P*=0.217）；而转染组细胞在G_2_期/M期所占比例为58.7%，空载体组为31.30%（*t*=4.560, *P*=0.010）。

**结论:**

Nm23-H1在人肺腺癌A549细胞的核内过表达使细胞主要分布在G_2_期/M期并促进了细胞的体外增殖。

## Abstract (en)

**Background and objective:**

Recent studies have indicated that Nm23-H1 is found in the nucleus, but previous studies have been based on the overexpression or suppression of Nm23-H1 in the cytoplasm. Due to the lacking nuclear localization signal of Nm23-H1, these results cannot reflect or repeat cells in which Nm23-H1 mainly positioned in nuclei and whether they cause clinical biological effects. Therefore, to explore the effects of transposing Nm23-H1 from the cytoplasm to the nucleus during lung cancer cell proliferation, a vector with a nuclear localization signal of Nm23-H1 was constructed and A549 cells were transfected.

**Methods:**

Gene recombination technology was used to construct pLentis-CMV-NME1-IRES2-PURO lentiviral vectors using a nuclear localization signal sequence, and the recombinant plasmid was verified using restriction enzyme analysis and sequencing. Nm23-H1 positioning and expression were performed after the stably transfected A549 cells were assessed by Western blot and confocal laser scanning microscope. The A549 cell proliferation was assessed using a cell counting kit-8. Flow cytometry was performed to assess the cell cycle distribution of A549 cells.

**Results:**

The directional Nm23-H1 lentiviral vector was successfully constructed within the nucleus. Compared with that of the empty vector group, the proliferation rates of the transfection groups at 72 h, 96 h, and 120 h were remarkably increased (*P* < 0.000, 1). Moreover, the empty vector group of A549 cells in the G_0_/G_1_ phase proportion was 35.69%, which was higher than the 28.28% of the transfection group (*t*=1.461, *P*=0.217); furthermore, the transfection group of A549 cells in the G_2_/M phase proportion was 58.7% and that of the empty vector group was 31.30% (*t*=4.560, *P*=0.010).

**Conclusion:**

Human lung adenocarcinoma cell line A549 cells of Nm23-H1 nuclear localized mainly in the G_2_/M phase and the nuclear Nm23-H1 promoted A549 cell proliferation *in vitro*.

肿瘤转移抑制基因*Nm23-H1*是第一个被分离鉴定出的与肿瘤转移相关的抑制基因。大量的基础研究表明，Nm23-H1能够逆转包括肺癌在内的多种恶性肿瘤的转移潜能。因此理论上其表达的高低与肺癌患者的预后应该成正相关。但越来越多的临床研究发现，Nm23-H1在肺癌的高表达并不都提示好的预后^[1]^。甚至有研究^[2]^发现其在鳞癌的表达反而与预后负相关。张志敏等^[3]^通过免疫组化研究发现在30例手术切除肺癌标本中，24例标本Nm23-H1的表达都以胞浆表达为主，但有6例标本存在Nm23-H1的胞核表达，而这6例患者均较早的出现局部复发或远处转移。同样在Kim等^[4]^的研究中也发现在头颈部鳞癌中，20%的患者存在Nm23-H1的胞核表达，而核高表达的Nm23-H1与较差的预后（OR=7.48）以及疾病的早期复发（OR=3.02）相关，这提示Nm23-H1除具有抑制肿瘤转移的抑癌基因特性外，可能还具有癌基因潜能，而这两种特性可能与Nm23-H1在胞浆胞核不同的定位有关。现有的基础研究均以过表达或抑制胞浆Nm23-H1为研究手段，研究结果并不能真实反映或重复临床中Nm23-H1以胞核定位为主细胞的实际生物学效应。因此，本研究构建了核内定向表达载体pLentis-CMV-NME1-IRES2-PURO，转染人肺腺癌A549细胞后观察细胞增殖变化，以期探讨胞核Nm23-H1对肺癌细胞增殖的影响。

## 1 材料和方法

### 1.1 主要材料与试剂

人肺腺癌A549细胞株由第三军医大学大坪医院野战外科研究所肿瘤中心保存并传代培养；大肠杆菌*E. coli* DH5α、293FT细胞由本实验室保存；DMEM培养基、小牛血清购自Gibco公司，胎牛血清购自Life Technologies公司；pLentis-CMV-IRES2-PURO载体购自美国Invitrogen公司产品；质粒提取试剂盒、胶回收试剂盒购自Omega公司；限制性内切酶、DNA聚合酶购自Takara公司；DNA连接酶购自Promega公司；兔抗人Nm23-H1抗体、兔抗人β-tublin购自美国Santa Cruz公司；山羊抗兔二抗购自北京中杉金桥生物技术有限公司；兔抗人Lamin A/C购自Bioworld Technology公司；CCK-8（Cell counting kit-8）购自碧云天生物技术研究所；Annexin V-FITC凋亡检测试剂盒购自北京大学人类疾病基因研究中心。

### 1.2 细胞培养

A549细胞用含10%小牛血清、链霉素（100 μg/mL）和青霉素（100 U/mL）的DMEM培养基，37 ℃、5%CO_2_条件下培养，每3天传代1次。0.25%胰酶常规消化，选择生长良好的对数期细胞进行实验。

### 1.3 Nm23-H1（NME1）表达载体构建、慢病毒包装及细胞转染

根据Nm23-H1设计PCR引物以扩增Nm23-H1（NME1）CDS序列。上游引物：TTAGGATCCaccATGaagcgacctgccgccacaaagaaggctggacaggctaagaagaagaaa ATGGCCAACTGTGAG；下游引物：GCACTCGAGTTAAGCATAATCTGGAACATCATATGGATATTCATAGATCCAGTTCT。其中上游引物带有*Bam*HI酶切位点以及核定位信号NLS序列，下游引物带有*Xho*I酶切位点以及HA tag序列。反应条件为：98 ℃、1 min，98 ℃、10 s，60 ℃、20 s，72 ℃、180 s，30个循环后，72 ℃延长3 min。PCR产物胶回收，获得回收产物NME1，载体连接后，感受态转化，提取质粒，按OMEGA质粒提取试剂盒说明书操作，用*Bam*HI酶切验证，正确的质粒送测序，测序引物为：AACAACTCCGCCCCATTGAC。酶切鉴定及测序均验证均正确。在293FT的培养板中加入氯喹至终浓度25 μM，加入灭菌水及以下质粒（pMD2.G 1.5 μg+pSPAX2 4.5 μg+pLentis-CMV-NME1-IRES2-PURO 6 μg），总体积为263 μL，然后加入2 mol CaCl_2_ 37 μL，混匀，最后再加入300 μL 2×HBS，边滴加边振荡，迅速将混合物加入到细胞培养孔中，轻轻摇晃混匀。将培养基换为2 mL新鲜DMEM培养基，收集培养皿中的上清，500 *g*离心10 min，然后将上清用0.45 μm滤器过滤，置于-70 ℃保存。将培养的目的细胞消化计数后按2×10^4^/孔铺于24孔板中，24 h后，分别加入对照病毒以及表达病毒，各250 μL，24 h后，弃去孔内培养基，加入新鲜的DMEM培养基500 μL，待细胞生长至80%融合度左右，将细胞消化并传入6孔板中，24 h后，吸取孔内培养基，换为含有合适浓度puromycin的DMEM培养基，待细胞长满后，消化收集细胞，穿入培养瓶中扩大培养。

### 1.4 蛋白质印迹法检测Nm23-H1的定位及表达

慢病毒稳定转染后的A549用细胞核/胞浆细胞组分提取试剂盒（Nuclear/Cytosol Fractionation Kit）分别提取胞浆蛋白及胞核蛋白。制作5%的浓缩胶和12.5%的分离胶，电泳后转至PVDF膜，然后使用封闭液（含15%脱脂奶粉的TBs液）封闭1 h，Nm23-H1抗体按1:1, 000比例稀释（稀释液为含0.05%Tween的TBS液），4 ℃孵育过夜。加标记了辣根过氧化物酶（herseradish peroxidase, HRP）的生物素二抗，按1:2, 000-1:4, 000比例稀释，室温轻摇孵育1 h，采用电化学发光法显影。

### 1.5 激光共聚焦检测Nm23-H1的定位及表达

分组处理A549细胞后移除培养基，取出已经贴壁的盖玻片，以0.01 mol的PBS冲洗，5 min一次，重复冲洗3次；2%甲醛/PBS溶液在室温条件下固定30 min，用PBS冲洗，冲洗步骤同前；加0.5% Triton X-100放置室温15 min，用PBS冲洗，冲洗步骤同前；以30 mL/L的山羊血清工作液于37 ℃封闭30 min，吸掉封闭液；在载玻片上滴加20 μL鼠抗人Nm23-H1单克隆抗体（稀释度1:1, 000）溶液，盖玻片细胞贴壁面与抗体接触，4 ℃孵育过夜；加入含有DAPI核染料于37 ℃孵育10 min。PBS冲洗同前，自然晾干切片，以缓冲甘油封片，以PBS替代一抗作为阴性对照；在Leica TCS SP激光共聚焦显微镜下观察和扫描，以软件ZEN 2012分析。

### 1.6 CCK-8法检测细胞增殖情况

A549细胞经nNME1（Nm23-H1核过表达）或Control-nNME1转染培养12 h-24 h后，同A549细胞一起参照CCK-8试剂盒说明，取2, 000个/孔接种至96孔培养板，分别培养24 h、48 h、72 h、96 h和120 h后弃去上清液，每孔加入含10 μL CCK-8的无血清培养液，再继续培养2 h后转移培养上清液至96孔平底比色板，根据调零孔调零，用酶标仪在450 nm波长下测定各孔吸光度（OD）值，绘制细胞增殖曲线。每组平行做5个复孔，实验重复3次。

### 1.7 细胞周期分析

细胞周期检测管加入80%冷乙醇固定后4 ℃过夜，冷PBS冲洗去除固定液，离心弃上清，加入300 μL PBS，10 mg/mL的核糖核酸酶A（RNase A）溶液15 μL混匀，37 ℃孵育30 min，加1 mg/mL的PI染液混匀，暗室中放置30 min后转入流式管进行荧光检测。采用ModFit LT软件分析细胞周期各时相的特点。

### 1.8 统计学方法

采用SPSS 19.0进行数据分析。每项实验均独立重复3次，结果以Mean±SD表示，各组间结果数据进行单因素方差分析，组间两两比较采用*t*检验分析并作图。以*P* < 0.05作为差异有统计学意义。

## 2 结果

### 2.1 核内定向表达载体pLentis-CMV-NME1-IRES2-PURO的构建及转染

重组载体质粒酶切鉴定及测序结果：质粒用*Bam*HI酶切验证，切出3, 400 bp及2, 200 bp的片段，所用Marker为Takara的1, 000 bp DNA Ladder（图 1），正确的质粒送测序，测序引物为CMV-F：AACAACTCCGCCCCATTGAC，测序均验证正确。载体示意图如下：pLentis-CMV-NME1-IRES2-PURO（图 2）。采用蛋白质印迹及激光共聚焦检测其Nm23-H1蛋白的定位及表达变化。蛋白质印迹及激光共聚焦结果显示未转染组和空载体组Nm23-H1蛋白以胞浆表达为主，而转染组（A549 nNME1）所标记了的在胞浆胞核都有表达，但主要以胞核表达为主，表明我们成功构建并转染了载体pLentis-CMV-NME1-IRES2-PURO，Nm23-H1蛋白在A549细胞核内成功过表达（图 3）。

**1:**
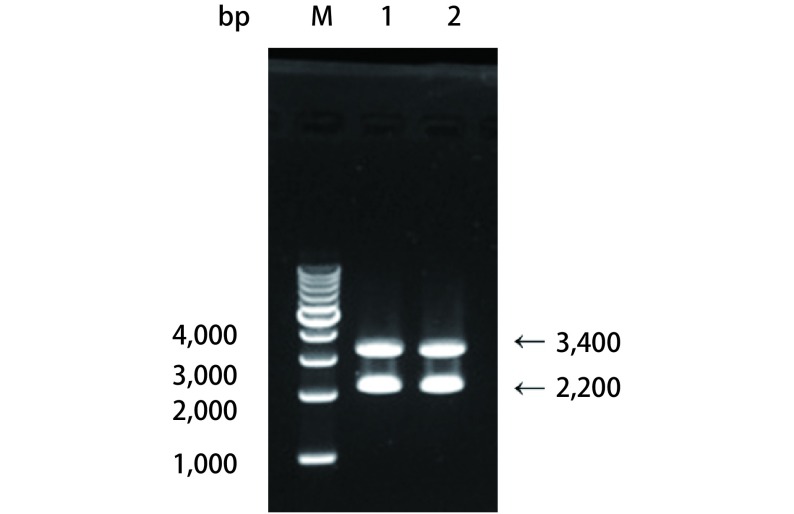
重组质粒pLentis-CMV-NME1-IRES2-PURO酶切鉴定结果 Results enzyme digestion analysis of recombinant plasmid. pLentis-CMV-NME1-IRES2-PURO. M: 1, 000 bp DNA Marker. Lane 1-2: pLentis-CMV-NME1-IRES2-PURO

**2:**
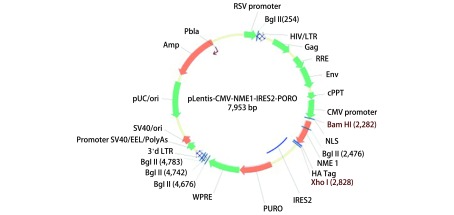
pLentis-CMV-NME1-IRES2-PURO表达载体 Recombinant plasmid pLentis-CMV-NME1-IRES2-PURO

**3:**
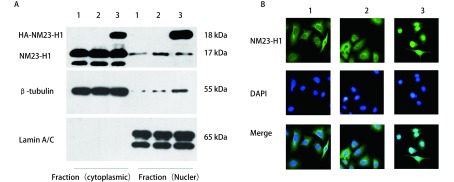
载体pLentis-CMV-NME1-IRES2-PURO转染后A549细胞中Nm23-H1蛋白的定位及表达。A：Nm23-H1蛋白在A549细胞中以胞浆表达为主，转染组带有HA标签的Nm23-H1蛋白在胞浆和胞核均检测到，但主要以胞核表达为主。*β*-tublin和Lamin A/C作为内对照；B：Nm23-H1蛋白主要位于A549细胞的胞浆，转染后其在细胞核表达明显增多；1：A549；2：A549空载体组；3：A549 nNME1转染组 Nm23-H1 positioning and expression after stably transfected A549 cells by pLentis-CMV-NME1-IRES2-PURO. A: Nm23-H1 was mainly localized in the cytoplasm of A549 cells, Nm23-H1 with HA tag was detected in cytoplasm and nucleus of A549 nNME1 transfected and mainly localized in nucleus. *β*-tublin and Lamin A/C served as an internal control; B: Nm23-H1 was mainly localized in the cytoplasm of A549 cells and its nuclear localization significantly increased after transfected; 1: A549; 2: A549 vector only transfected; 3: A549 nNME1 transfected

### 2.2 Nm23-H1核内定向表达对A549细胞增殖的影响

CCK-8法检测结果显示，空载体组与空白对照组相比，二者增殖速度相当，无明显差异，Nm23-H1核过表达组增殖速度快于对照组和空载体组，与空载体组相比在72 h（*t*=6.223, 6, *P* < 0.000, 1）、96 h（*t*=11.501, 8, *P* < 0.000, 1）和120 h（*t*=10.818, *P* < 0.000, 1），差异有统计学意义（图 4）。

**4:**
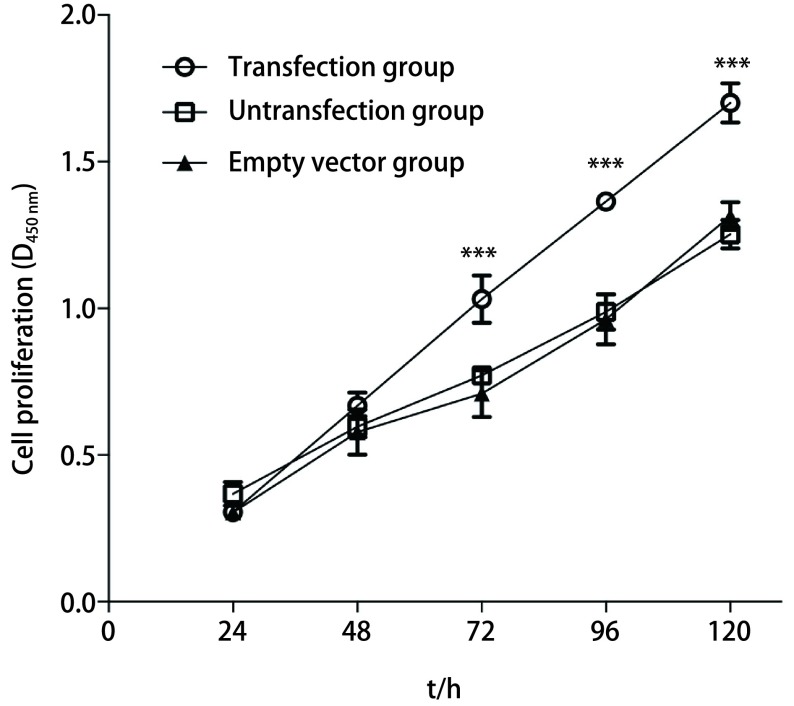
CCK-8法检测Nm23-H1核过表达对A549细胞增殖的促进作用。***P < 0.000, 1，*n*=3 The nuclear Nm23-H1 promotes A549 cells proliferation in vitro was detected by cell counting kit-8 (CCK-8). ***P < 0.000, 1, *n*=3

### 2.3 Nm23-H1核内定向表达对A549细胞周期的影响

流式细胞仪分析结果显示，空载体组A549细胞G_0_期/G_1_期所占比例为35.69%，高于A549 nNME1转染组的28.28%，差异不具有统计学意义（*t*=1.461, *P*=0.217）；而转染组细胞G_2_期/M期百分比为58.7%，空载体组A549细胞百分比31.30%，差异具有统计学意义（*t*=4.560, *P*=0.010）；空载体组A549细胞S期所占比例为29.53%，转染组细胞S期所占比例为11.21%，差异有统计学意义（*t*=4.427, *P* =0.011）。提示空载体组细胞主要分布在G_0_期/G_1_期，而转染组细胞则主要分布于G_2_期/M期（表 1）（图 5）。

**1:** Nm23-H1核内定向表达对A549细胞周期的影响（mean±SD, *n*=3） Nm23-H1nuclear directional expression effect on A549 cell cycle (mean±SD, *n*=3)

Group	Cell circle distribution (%)		
	G_0_/G_1_	S	G_2_/M
Untransfected group	40.28±6.67	27.2±15.54	30.76±9.20
Empty vector group	35.69±8.75	29.53±2.81	31.30±6.38
Transfection group	28.28±0.72	11.21±6.59	58.7±8.22

**5:**
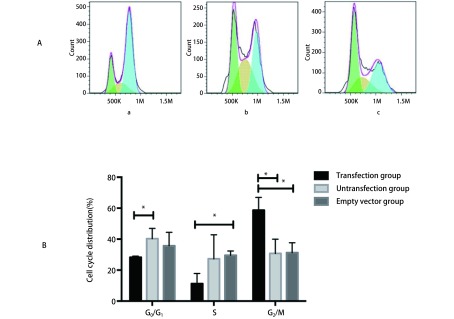
A549、A549空载体组和A549 nNME1转染组的细胞周期检测。A：A549、A549空载体组和A549 nNME1转染组细胞的流式细胞术周期检测结果，a：A549；b: A549空载体组；c：A549 nNME1转染组；B：转染组细胞在G_2_期/M期所占比例（58.7%）明显高于空载体组（31.30%）和未转染组（30.7%）。* *P* < 0.05 Changes in the level of cell cycle in A549, A549 vector only transfected and A549 nNME1 transfected group cells. A: The flow cytometry results showed the cell cycle of A549, A549 vector only transfected and A549 nNME1 transfected group cells; a: A549; b: A549 vector only transfected; c: A549 nNME1 transfected; B:The cell circle distribution of A549 nNME1 transfected (58.7%) was significantly higher than those of A549 vector only transfected (31.30%) and A549 cells (30.7%). *P < 0.05

## 3 讨论

肿瘤细胞的扩散及转移的发生、发展是一个多基因、多步骤的复杂过程，涉及抑癌基因表达下降、缺失或功能失活和相关癌基因的过量表达或功能失常。*Nm23-H1*作为转移抑制基因，对此过程的多个环节都具有调节功能。它能降低肿瘤细胞的运动力、侵袭力，同时促进细胞分化^[5]^。理论上其表达越高，肺癌的的预后越好，但临床实际情况并非如此。Wang^[1]^采用RT-PCR检测了141例非小细胞肺癌组织Nm23-H1 mRNA表达，发现Nm23-H1表达的高低与预后无关。而Gazzeri等^[2]^进一步发现鳞癌中Nm23-H1蛋白的高表达与肿瘤病程进展具有相关性，即表达越高，预后越差。这说明Nm23-H1在肺癌中可能存在双面效应，即抑癌基因效应与促癌基因效应。在其他肿瘤中，Li等^[6]^报道在老年喉癌患者中，Nm23-H1与预后较差以及疾病复发显著相关（*P*=0.000, 9）。同样在宫颈癌^[7]^以及血液肿瘤^[8]^的临床研究也发现，Nm23-H1的表达越高，预后越差。这进一步提示Nm23-H1存在双面效应的可能性。

Nm23-H1有可能存在双面效应，而这种双面效应可能与其胞浆胞核不同的定位有关。Subramanian等^[9]^报道EB病毒核抗原3C（EBNA3C）与Nm23-H1结合后，将促使Nm23-H1从胞浆转移至胞核，同时研究还发现Nm23-H1转位后，其抑制肿瘤转移的特性不仅被EBNA3C废除，反而还增加了EBNA-3C的转录活性。Kaul等^[10]^发现在EBNA3C蛋白诱导下进入胞核后，同样也表现为促进肿瘤生长效应。因此本研究首次以连接核引导信号肽的Nm23-H1构建在胞核定位为主的肺癌细胞系，即构建并转染了载体pLentis-CMV-NME1-IRES2-PURO，使Nm23-H1蛋白在A549细胞核内定向表达。此次核定位信号NLS序列aagcgacctgccgccacaaagaaggctggacaggctaagaagaagaaa，翻译的氨基酸为Lys、Arg、Pro、Ala、Ala、Thr、Lys、Ala、Gly、Gln、Ala、Lys、Lys、Lys、Lys，从而组成核定位信号肽。已知多数核蛋白是通过importin α/β异源二聚体运载系统被转运到细胞核内，以此方式入核的蛋白都有羧基端核定位信号肽（nuclear localization signal sequence, NLS），importin α可识别NLS，并形成被运载蛋白-importin α-importin β的三联复合物，将入核蛋白转运至胞核内。我们此次的入核蛋白也有同样的核定位信号肽，但它是否是通过importin α堆运载系统入核，还尚无定论。细胞增殖实验表明，Nm23-H1核过表达的A549细胞在72 h、96 h和120 h时增殖率均大于空载体组的细胞增殖率，且有统计学意义。因此研究结果表明Nm23-H1核过表达对人肺腺癌A549细胞不再是抑癌作用，相反以促癌效应为主。这一结果与单纯胞浆过表达Nm23-H1蛋白是不同的，也是本研究的意义所在，证明了入核后的Nm23-H1蛋白可能起到了其同源异构体Nm23-H2的癌基因潜能作用^[6]^。

既往研究表明，在人肺腺癌A549细胞胞浆中过表达Nm23-H1蛋白，可抑制肿瘤细胞的增殖，使G_1_期细胞增加而S期细胞减少，停滞于G_0_期，从而抑制体外培养的A549肿瘤细胞的增殖^[11]^。我们早期研究也发现，紫杉酵脂质体对Nm23-H1 siRNA转染A549细胞周期的影响提示Nm23-H1低表达组在紫杉醇脂质体作用于主要将细胞阻滞于G_2_期/M期，以增强紫杉醇脂质体对肿瘤细胞的抑制和杀伤作用^[12]^。以上研究表明胞浆过表达Nm23-H1蛋白对于人肺腺癌A549细胞的周期影响是主要将细胞阻滞在G_0_期/G_1_期，从而抑制肿瘤细胞增殖，而在紫杉酵脂质体作用下的A549细胞则是因Nm23-H1的低表达被阻滞于G_2_期/M期而抑制细胞增殖。本研究发现在胞核过表达Nm23-H1蛋白，空载体组细胞主要分布在G_0_期/G_1_期，而转染组细胞则主要分布在G_2_期/M期。本研究中Nm23-H1核内的定向表达组细胞则主要分布在G_2_期/M期，与主要分布在G_0_期/G_1_期空载体组相比，促进了细胞的增殖。说明Nm23-H1可能主要通过影响细胞周期来产生促癌效应。

目前已有研究提示Nm23-H1具有促进肿瘤细胞增殖、转录调控活性及潜在的促癌作用，但大部分来源于病毒癌蛋白的作用^[9, 13]^，且至今尚缺乏直接证据。曾有研究者分析认为，Nm23-H1之所以不再具有抑制肿瘤转移的特性及促生长作用，其原因可能是由于病毒致癌蛋白与Nm23-H1的结合封闭了Nm23-H1的激酶活性位点，影响其在胞浆胞核的分布。其详细的作用机制有待进一步研究。

综上所述，本研究成功构建核内定向表达载体pLentis-CMV-NME1-IRES2-PURO，发现了人肺腺癌核内定向表达Nm23-H1使细胞主要分布在G_2_期/M期并促进了A549细胞的体外增殖。但核内定向表达Nm23-H1以直接或间接的方式激活下游与增殖相关基因的表达，从而发挥促进肺癌增殖的作用途径和作用机制尚待进一步研究阐明。
